# Medical and Philosophical Intelligence

**Published:** 1815-02

**Authors:** 


### MEDICAL AND PHILOSOPHICAL INTELLIGENCE.

ROYAL SOCIETY.—On Wednesday, the 30th of November, the day of the anniversary election, the Copleyan medal was given to Mr. IVORY for his Mathematical Papers published in the Transactions. The following officers were elected for the ensuing year:—

*President*,—Right Hon. Sir Joseph Banks, Bart. K. B.

*Secretaries*,—William Hyde Wollaston, M. D., Taylor Combe, Esq. M. A.

*Treasurer*,—Samuel Lysons, Esq.

*Of the Old Council.*—Right Hon. Sir Joseph Banks, Sir Charles Blagden, Knt., Samuel Goodenough, Lord Bishop of Carlisle, V. P., Taylor Combe, Esq. Secretary, Samuel Lysons, Esq. Treasurer, V. P., George Earl of Morton, K.T. V.P., Thomas Murdoch, Esq., John Pond, Esq. Astr. Royal, William Charles Wells, M.D., William Hyde Wollaston, M. D. Secretary, Thomas Young, M.D. Secretary for Correspondence.

*Of the New Council.*—Mr. William Allen, William Blake, Esq., Rev. C. Burney, LL.D., Charles William Earl of Charleville, Davies Giddy, Esq. M. P., Sir Everard Home, Bart., James Horsburgh, Esq., Alexander Marcet, M.D., Thomas, Earl of Selkirk, Henry Warburton, Esq.

On Thursday, Dec. 15, part of a paper by BENJAMIN TRAVERS, Esq. was read, On the Mechanism by which the Eye adjusts itself to different Distances. It consisted of three parts: 1. A sketch of the different hypotheses to which this mechanism has been ascribed, with the author's reasons for not admitting them. These are the muscles of the eye, the ciliary processes, the supposed muscularity of the lens itself. 2. An account of the anatomy of the eye, as far as it is requisite for the purposes of the paper. 3. His reasons for believing that external pressure is applied to the lens, and that this pressure is sufficient to alter its shape.

On Thursday, 22d Dec. Mr. Travers's paper was continued. He gave his reasons for considering the iris as muscular, and that by means of it the pupil is adjusted to different distances.

---

Mr. WARD has requested us to insert the following *Report of the General Committee of Associated Apothecaries, &c. held at the Crown and Anchor, January 10th*, 1815.—This Committee having now received a printed copy of the "Bill for enlarging the Charter of the Society of Apothecaries in the City of London, granted by his Majesty King James the First, and for better regulating the Practice of Apothecaries throughout England and Wales," which is intended to be introduced into parliament in the present session, have carefully examined the same.

The committee learn, with sincere concern, that the Royal College of Physicians have not admitted of any alteration or modification of the clause, making it penal for any apothecary, refusing to compound compound or unfaithfully compounding medicines prescribed, notwithstanding the memorial of this committee, dated May 25th, 1814, to that effect.

Consequently, the committee deem it to be their duty to petition against the Bill, merely that they may be entitled, when the Bill is submitted to a committee in parliament, to appear there by counsel for obtaining such modification of the said clause as they have been instructed to propose; and, likewise, that they may have opportunity to watch over the important interests confided to their charge.

The committee have great satisfaction in remarking, that all the most material suggestions resulting from the general meeting of May 12th ult. or since offered by the committee, have been freely and readily adopted by the Committee of the Society of Apothecaries, who appear actuated by a real desire for the passing of an Act, as consonant with the wishes of every apothecary, as conducive to the improvement and interests of that branch of the medical profession.

The committee have ordered the said Bill to be printed, and a copy to be sent to the chairman or secretary of every district meeting; and that the following resolution be published.

"It appearing to this committee, that several of the country districts have expressed their disapprobation of the Bill now about to be introduced into parliament, from its not including the practice of surgery and midwifery.

"Resolved, That such districts be informed, that these branches of the medical profession could not be embodied into the Act 'for better regulating the Practice of the Apothecary;' but the committee trust, if this Act should receive the sanction of the legislature, that the Royal College of Surgeons will think it right to apply to the same authority for power to superintend the regulation of the practice of surgery and midwifery. If, however, that body should not undertake such application to parliament, the committee will consider it their duty, on the behalf of the Surgeons and Apothecaries of England and Wales, to adopt such measures as they may be advised, to bring in a bill for the accomplishment of that object."

G. M. Burrows, Chairman.

---

A new method of operating for the cure of Popliteal Aneurism has been employed in Dublin with the most complete success.

The operation was performed by Mr. Crampton, Surgeon-General at the King's Military Infirmary, near Dublin, on the 15th of October. "The femoral artery was laid bare at the usual place, by an incision three inches in length and *compressed*, (but not tied circularly,) by a narrow tape, so as completely to obstruct the current of the blood *with the least possible disturbance to the artery*, or *injury to its coats.* The ligature was, by a peculiar contrivance, applied in such a manner as to enable the operator to tighten or to relax it at pleasure, without interfering with the wound. In two hours and a quarter the ligature was gently relaxed, but not completely loosened; no pulsation in the ham.

In

In twenty-four hours the artery was relieved from all compression; but, as a measure of precaution, the ligature was left in the wound. In forty-eight hours the ligature was withdrawn, and the wound was united by adhesive plaster."

The patient was examined on the fifth day by several professional gentlemen; his health was not in the least degree deranged. The tumor, which had decreased by one half, was without pulsation, and nearly incompressible. The temperature of each foot was 84°.

On the 14th day the wound was nearly healed, and the man went about the ward on crutches.

On the 18th day the wound was healed, and the tumor which could be seen only in the extended position of the limb, was incompressible, and altogether free from pain.

The advantage of Mr. Crampton's operation consists in its imitating in the most favourable way the process of nature, in the spontaneous cure of aneurism.

1st. By interrupting the course of the blood through the ruptured artery, the fluid contents of the aneurismal sac are allowed to coagulate, and the circulation is thrown upon the collateral branches.

2d. The subsequent obliteration of the artery is effected by a natural process, which protects the patient from the long train of sufferings, and of dangers necessarily attendant upon the permanent contraction of a great artery, and its separation from the ligature by the process of sloughing or ulceration.

---

*Magnesia in Human Bones.*—Fourcroy and Vauquelin first discovered the presence of magnesia in bones of inferior animals; but they could detect none in human bones. For this they assigned a physiological reason. In man the magnesia is carried off in the urine; but this is not the case in the inferior animals. Hence it appears in the bones of the latter, but not of the former. Berzelius repeated the experiments of Fourcroy and Vauquelin, and could not by their method detect any magnesia in human bones; but he gives another process, by which he assures us he detected it. Professor Hildebrandt, of Erlanger, has lately resumed this subject, and repeated the experiments of Fourcroy and Vauquelin. Like them, he could detect no magnesia in human bones, and therefore concludes that Berzelius was mistaken. Dr. Thompson acknowledges that this mode of proceeding appears very extraordinary: to repeat a process, which, Berzelius himself admits, does not yield any magnesia, and then to conclude that Berzelius is mistaken because the experiment turns out just as he foretold it would. To refute Berzelius' conclusion, it would be necessary to repeat the experiment as he describes it, or to show experimentally that the experiment is incapable of repetition.

---

*Urine.*—Professor Wurzer has published a set of experiments on a remarkable urine emitted by a man of the age of 33. He had been been afflicted with a gonorrhæa virulenta ever since the age of 24. In consequence of cold, to which he had been exposed, his breasts swelled, and a few days after he passed the remarkable urine which drew the attention of Professor Wurzer. It was milk-white, and contained in it no inconsiderable quantity of a matter which possessed exactly the properties of *curd*.

---

Mr. CARPUE, of Dean-street, is the first person in England who has adopted the East Indian operation for remedying the deformity of a lost nose. An officer of distinction lost his nose in actual service in Egypt, though otherwise enjoying robust health. On his return home, hearing that Mr. Carpue had, in his Anatomical Lectures, mentioned the possibility of supplying the defect; and that the story of Taliacotius was not a fable, there being a very beautiful work of his on the subject, in which the whole process was described, and it being known that in India one of the casts of the natives was in the habit of performing this operation, applied to that gentleman, who readily undertook the experiment. The consequence was, that by bringing down a piece of the flesh and skin from the forehead, he has completely succeeded in forming a new nose, which partakes of the regular circulation of the blood, and is not to be distinguished from the original. The forehead has also recovered the flesh and skin which were cut out to supply the deficiency.

---

Some late Numbers of the Monthly Magazine contain an account of relief to consumptive patients from inhaling the effluvium of fresh-dug earth, particularly under green sod. There are also some remarks on the antiseptic properties of air thus evolved. We have not transcribed them, because they are not sufficiently correct in the pathological part of the inquiry for the readers of a medical work. The hints they furnished may, however, be improved.

---

In a periodical journal, we meet with a recipe for chilblains, which seems to promise success, and which is at least worth trying in the early part of the winter. It consists of the addition of a small portion of cantharides to the volatile liniment or *linimentum ammoniæ*.

---

Dr. JARROLD, of Manchester, remarks that distorted shapes, though usually considered as the effect of scrofula or injuries, are for the most part only to be accounted for by some original predisposition. He is preparing a work on that subject. We recommend him in his intended publication to define with accuracy what he means by scrofula. Accidents to which these cases are generally ascribed by parents, are very improbable causes, because an accident which would induce such an effect suddenly, would probably destroy the patient; but sure Dr. Jarrold must be aware how frequently the distorted spine is accompanied with enlarged mysenteric glands, and other diseases usually designated by the vague expression of scrofula. From the ingenious author of

Anthropologia

Anthropologia we expect much information, and shall be greatly disappointed if we do not meet with equal accuracy. We shall be ready to make our pages useful for any previous discussion on the subject, conducted with that propriety which always attends well-informed writers with the best intentions.

---

The extract of Lactuca *Virosa*, the mode of whose preparation may be found in Collins's Observationes Circa Morbos, is recommended by a German physician as a remedy for Pertussis, which has not only been found successful in his own hands, but has the testimony of men of considerable eminence in its favour. The cases related of its successful employment do not lead us to be very sanguine, but it is at least deserving of trial. A child of two years may take half a grain three times a day; the dose must, however, be regulated according to its effects. This physician divides Pertussis into a catarrhal and a convulsive stage, and considers the remedy to be only applicable in the latter. It is said to be more antispasmodic and diuretic than preparations of the other species of Lactuca, which it is of consequence to observe, as they are frequently substituted for it.

---

The neighbourhood of Paris has been visited by a severe epezootic among the horned cattle. A report has been given to the faculty by Mr. Hurard, of the National Institute. Though the mischief has not been so considerable as was at one time suspected, yet we are anxious to give a faithful abstract of the paper, as it contains a great quantity of valuable information which may be highly serviceable, should such a calamity ever return among us.

It appears, beyond contradiction, that the disease was imported into France with the hostile and allied armies, and seemed to originate among the cattle attached to the troops by the same means as the camp fever among the soldiers. But it is necessary to remark, that the infection does not spread from cattle to men. This is not easily ascertained in a camp, where man, in common with all the other animals, engaged in the campaign, is often affected with disease, but it has invariably been found that none of the peasantry have suffered when their whole herds have been destroyed by the temporary requisition of their stalls or muck yards, for the camp cattle during the marches of the armies.

The following are the most important paragraphs in the *Résumé.*

"It has always been imported into France by contagion, and that contagion has been owing to animals who have been sent out of their own country, fatigued, heated, badly driven, and badly fed, from which causes the disease has been spontaneously excited or originated without previous contagion. Whatever name may have been given to this disease, it is easily detected by the external symptoms, and on opening dead animals.—Its character is inflammation of the abdominal viscera, principally of the stomach's internal canal and liver, whence have occurred dysenteries and bilious diarrhœa. The allied troops ate of the flesh of animals affected with this complaint before their arrival in France; all Paris and its environs, all the troops who occupied it and surrounded it, were fed during two months on this meat, even the sick at the hospitals made use of it. The number of invalids was not increased by it, nor did it occasion any epidemic among the troops or the people, and, during this time, a typhus disappeared which had prevailed before the epizootic.

"There is little reliance to be placed on curative treatment, notwithstanding the pretended success announced in some writings, because a very small number of animals were cured; because some recovered without any treatment, by the efforts of nature only, and we must not attribute to the first that which is due only to the last.

"Although the preservative (*preservatif*) treatment is not always infallible, still it is upon that we can more justly reckon; it consists in seasonable blood-letting, in setons, in bathing and frequent washing with cold water, in the use of bitters and acid minerals, in exposing the animals to a free circulation of air, frequent dry rubbing all over the body, fumigation of muriatic oxigenated acids, &c.

"The most essential part of this treatment, (the only part from which we have any right to expect certain results) is that which is the most generally neglected, viz. the entire separation of the healthy animals from the diseased. This separation is the more necessary as the disease is so highly contagious, and as the contagion is propagated by every means of communication."

On the above paper, we cannot withhold a few remarks: first, that the disease was evidently the *typhus castrensis;* and, therefore, we are not certain its origin was similar to the diseases which have appeared among that race of animals in England, which last may have arisen from some constitution of the atmosphere. Secondly, that, though the cattle were ill fed, ill treated, and overdriven, their danger arose entirely from high inflammation, which was more rapid in its progress than it usually is in the best fed of the human race. There is, indeed, a remark to this point in the Résumé: *Elle est generalement mortelle comme le sont toutes les fortes inflammations dans les herbivores.*

That as far as analogy can direct us, we have a confirmation of the propriety of free blood-letting in those camp fevers, which have generally been termed typhoid, and for which we have too long persevered in a stimulant on tonic practice.

---

We have received Paris Journals, but no later than to the end of November. The following is the only extract we have room for in the present number. They contain also abstracts of two very good communications in the North American Journals. These we shall give in our next number, unless the originals should arrive first.

*An Account of a Cæsarian Operation, followed by the most complete success.* By M. Charmeil, Ex-Surgeon-in-Chief to the Army, &c.—A woman, forty years of age, badly formed, had been in labour for a week, when Mr. Charmeil was sent for; he learnt, that in four preceding pregnancies, she could not be delivered but by the mutilation of the infants. He estimated the

 diameter diameter of the pelvis from its anterior to its posterior brim, 15 or 16 lines, giving the superior opening the form of the figure 8.

Despairing of the possibility of terminating the delivery by natural means, he proposed the Cæsarian operation, to which the woman submitted chearfully. The incision was made obliquely at the extremity of the rectus muscle (*sterno pubien*) on the right side, towards which the bottom of the uterus was inclined. A spirting of blood, which appeared when the uterus was cut, stopped directly by the contraction of that viscus. An assistant retained, with his hands, the intestines which were in danger of escaping by the wound. A living child and the placenta were easily extracted. The wound of the abdominal parietes was re-united by three stitches, and covered by a defensive apparatus. Mr. Charmeil was obliged to be absent, and did not see his patient again till the 19th day, when he found her almost well and suckling her infant. Fifteen months afterwards, he again saw her, when she had a ventral hernia, which she easily kept up by a napkin, and which did not prevent her daily occupation. The following year, this woman was pregnant again, she submitted a second time to the Cæsarian operation; a different surgeon operated, following the traces of the former cicatrix. Mr. Charmeil thinks it more adviseable to make the incision along the edge of the rectus muscle than the linea alba, because the subject is less exposed to hemorrhage, and it is by these means those fluids, which ought to come out by the incision, more readily escape.

---

Mr. Nicholus will commence his first Course of Lectures on the Natural History, Structure, and Formation of the Teeth, at six o'clock in the evening of Friday, the 24th of February. During which, he purposes considering the Diseases incident to the Mouth, and describing the various operations necessary for their removal.

---

*Lectures and Examinations on Physic, Chemistry, and the Materia Medica*, by Dr. Hooper and Dr. Ager, at the Theatre of Mr. Brookes, Blenheim-street, Great Marlborough-street.—The next Course will begin on Monday, February 6, 1815, at 8 in the morning.

The Course will comprehend an explanation of,

1. The best method of destroying the different kinds of infection.

2. The most approved way of treating persons who have taken poison, or been exposed to noxious exhalations, with the means of detecting them chemically, and obviating their pernicious effects.

3. The phænomena of galvanism and electricity, and their application to the cure of diseases.

*** The Lectures on the Theory and Practice of Physic will be delivered every morning (Sundays excepted) at eight o'clock; and those on Chemistry and the Materia Medica at nine. Three Courses are given yearly, beginning in February, June, and October, each on the first Monday of the month.

### METEOROLOGICAL REGISTER.

*From December the 25th,* 1814, *to January the 25th,* 1815.

Kept by C. BLUNT, Philosophical Instrument Maker, No. 38, Tavistock-Street, Covent-Garden.

| Moon. | Day. | Wind. | Barometrical Pressure. Max. | Min. | Mean. | Temperature. Max. | Min. | Mean. | |
|---|---|---|---|---|---|---|---|---|---|
| ○ | 26 | NE | 29·59 | 29·5 | 29·545 | 38 | 31 | 34 | Snow&Rain |
| | 27 | SE | 29·4 | 29·2 | 29·267 | 37 | 33 | 34·3 | Rain |
| | 28 | SE | 29·4 | 28·99 | 29·235 | 46 | 31 | 37·3 | Rain |
| | 29 | SE | 29·73 | 29·69 | 29·705 | 48 | 35 | 40·3 | Rain |
| | 30 | E | 29·8 | 29·7 | 29·737 | 49 | 36 | 43·3 | Rain |
| | 31 | N | 30·04 | 29·94 | 29·98 | 47 | 33 | 40 | Fair |
| | 1 | N | 30·3 | 30·3 | 30·3 | 39 | 32 | 35·3 | Fair |
| ◑ | 2 | N | 30·38 | 30·36 | 30·37 | 38 | 30 | 33·6 | Fair |
| | 3 | N | 30·34 | 30·22 | 30·277 | 37 | 30 | 33·3 | Fair |
| | 4 | N | 30·12 | 30·04 | 30·063 | 36 | 30 | 33 | Fair |
| | 5 | N | 30·03 | 30·01 | 30·016 | 35 | 29 | 32·3 | Fair Snow |
| | 6 | NE | 30·01 | 29·99 | 30·005 | 33 | 24 | 32·6 | Snow at night |
| | 7 | NW | 29·92 | 29·68 | 29·76 | 34 | 26 | 28·6 | Fair Rain |
| | 8 | NW | 29·74 | 29·74 | 29·74 | 35 | 25 | 29 | Fair |
| | 9 | NW, SW | 29·97 | 29·8 | 29·907 | 35 | 28 | 35·3 | Fair |
| ● | 10 | SW | 29·77 | 29·75 | 29·765 | 43 | 37 | 36·6 | Fair |
| | 11 | SW | 29·73 | 29·59 | 29·65 | 40 | 33 | 39 | Rain Wind |
| | 12 | NW | 30·06 | 30· | 30·029 | 40 | 26 | 33·6 | Fair |
| | 13 | N | 30·08 | 29·8 | 29·832 | 36 | 28 | 32·3 | Fair |
| | 14 | NW | 29·82 | 29·76 | 29·785 | 35 | 29 | 32 | Fair |
| | 15 | NW | 29·82 | 29·82 | 29·82 | 34 | 27 | 31·3 | Rain |
| | 16 | E | 30·2 | 30·19 | 30·186 | 32 | 27 | 29·3 | Rain |
| | 17 | E | 30·23 | 30·22 | 30·227 | 33 | 30 | 28·6 | Snow |
| ◐ | 18 | E | 30·1 | 30·05 | 30·062 | 32 | 29 | 28·3 | Snow |
| | 19 | E | 29·79 | 29·79 | 29·79 | 32 | 28 | 27·6 | Snow |
| | 20 | E | 29·95 | 29·83 | 29·87 | 32 | 24 | 26 | Snow |
| | 21 | E | 29·77 | 29·76 | 29·762 | 32 | 25 | 29 | Snow |
| | 22 | NE | 29·78 | 29·78 | 29·78 | 32 | 25 | 28·3 | Snow |
| | 23 | NE | 29·82 | 29·80 | 29·81 | 29 | 19 | 29 | Fog |
| | 24 | NE | 29·70 | 29·52 | 29·59 | 29 | 20 | 23 | Fog & Snow |
| | 25 | NE | 29·59 | 29·59 | 29·59 | 28 | 19 | 22·6 | Fog |

RESULTS.

Mean barometrical pressure of the month 29·6598
Maximum 30·38 wind at N
Minimum 29·20 ——— SE

Mean temperature of the month 32·15 deg.
Maximum 49, wind at E
Minimum 19, ——— NE

*Scale exhibiting the prevailing Winds during the Month.*

| N | NE | E | SE | S | SW | W | NW |
|---|---|---|---|---|---|---|---|
| 7 | 6 | 7 | 3 | 0 | 3 | 5 | 0 |

| | Mean barometrical pressure. | Mean temperature. |
|---|---|---|
| From the full moon on the 26th Dec. to the last quarter on the 2d Jan. | 29·681 | 36·78 |
| ——— last quarter on the 2d, to the new moon on the 10th | 29·892 | 31·96 |
| ——— new moon on the 10th, to the first quarter on the 18th | 29·911 | 32·83 |
| ——— first quarter on the 18th, to the full moon on the 25th | 29·813 | 27·31 |

General

### *General Bill of Mortality, from Dec.* 14, 1813, *to Dec.* 14, 1814, *as delivered to the King by the Company of Parish Clerks.*

| DISEASES. | |
|---|---|
| Abortive & Stilborn | 683 |
| Abscess | 71 |
| Aged | 1774 |
| Ague | 2 |
| Apoplexy & Suddenly | 335 |
| Asthma | 952 |
| Bedridden | 1 |
| Bile | 9 |
| Bleeding | 29 |
| Bursten & Rupture | 23 |
| Cancer | 81 |
| Chicken-pox | 1 |
| Childbed | 216 |
| Colds | 28 |
| Colic, Gripes, &c. | 21 |
| Consumption | 4829 |
| Convulsions | 3418 |
| Cough and Hooping Cough | 864 |
| Cramp | 1 |
| Croup | 85 |
| Diabetes | 1 |
| Dropsy | 782 |
| Eaten by Lice | 1 |
| Evil | 5 |
| Fevers of all kinds | 908 |
| Fistula | 5 |
| Flux | 10 |
| French Pox | 12 |
| Gout | 53 |
| Gravel, Stone, Strangury | 12 |
| Grief | 1 |
| Head-ach | 2 |
| Headmoldshot, Horseshoehead, & Water in the Head | 421 |
| Imposthume | 3 |
| Inflammation | 1008 |
| Influenza | 2 |
| Jaundice | 57 |
| Jaw Locked | 5 |
| Lethargy | 2 |
| Livergrown | 27 |
| Lunatic | 223 |
| Measles | 817 |
| Miscarriage | 1 |
| Mortification | 224 |
| Overgrown Head | 1 |
| Palpitation of the Heart | 6 |
| Palsy | 149 |
| Pleurisy | 19 |
| Quinsy | 6 |
| Rash | 1 |
| Rheumatism | 11 |
| Scurvy | 8 |
| Shingles | 1 |
| Small-pox | 638 |
| Sore Throat | 6 |
| Sores and Ulcers | 11 |
| Spasm | 33 |
| St. Anthony's Fire | 4 |
| Stoppage in the Stomach | 25 |
| Surfeit | 2 |
| St. Vitus's Dance | 1 |
| Swelling | 1 |
| Teeth | 406 |
| Thrush | 68 |
| Tumor | 4 |
| Tympany | 1 |
| Water in the Chest | 35 |
| Worms | 1 |

| CASUALTIES. | |
|---|---|
| Bit by mad Dogs | 2 |
| Broken Limbs | 4 |
| Bruised | 4 |
| Burnt | 35 |
| Drowned | 111 |
| Excessive Drinking | 10 |
| Executed | 10 |
| Found Dead | 24 |
| Fractured | 2 |
| Frighted | 3 |
| Frozen | 2 |
| Killed by a Stag | 1 |
| Killed by Falls & several other Accidents | 83 |
| Killed themselves | 24 |
| Murdered | 5 |
| Overjoy | 1 |
| Poisoned | 5 |
| Scalded | 6 |
| Shot | 3 |
| Smothered | 1 |
| Starved | 1 |
| Suffocated | 11 |

| | | | |
|---|---|---|---|
| Christened | Males | 10313 | In all 20170 |
| | Females | 9857 | |
| Buried | Males | 10287 | In all 19783 |
| | Females | 9496 | |

Whereof have died,

| | |
|---|---|
| Under Two Years of Age | 5845 |
| Between Two and Five | 2038 |
| Five and Ten | 770 |
| Ten and Twenty | 649 |
| Twenty and Thirty | 1268 |
| Thirty and Forty | 1678 |
| Forty and Fifty | 1950 |
| Fifty and Sixty | 1810 |
| Sixty and Seventy | 1747 |
| Seventy and Eighty | 1343 |
| Eighty and Ninety | 592 |
| Ninety and a Hundred | 83 |
| A Hundred | 1 |
| A Hundred and One | 1 |
| A Hundred and Two | 1 |
| A Hundred and Eight | 1 |
| A Hundred and Eleven | 1 |

Increased in the Burials this Year 2461.

There have been Executed in the City of London 17; of which Number 10 only have been reported to be Buried within the Bills of Mortality.

The increase of burials has been, it may be observed, principally from inflammation and fever. Small-pox continues to lessen. Consumption is somewhat increased, and its attendants asthma and croup, croup, which may be attributed to the length and severity of the late winter.

The confined parts of the town appear still to be the most unhealthy. Within the walls of London, and in the ten parishes of Westminster, the burials exceed the christenings; whereas, in the seventeen parishes without the walls, and in the twenty-three out-parishes in Middlesex and Surry, the christenings exceed the burials very considerably.

---

### REPORT OF DISEASES.

During the last two months, catarrhal complaints have been frequent, and the family of winter coughs numerous, and, in some cases, distressingly severe. The most frequent form of pulmonic affection in the Reporter's practice has been simple cough, cough with dyspnœa, with hoarseness, and with hæmoptoe. In some instances, the hoarseness has continued troublesome after the cough has ceased. This is strikingly exemplified in an African, aged fifty years, who left Jamaica whilst only eighteen years old, and has since been engaged as a musician in the army. He was affected with very severe cough, and occasionally spat blood, though in small quantity; his strength was much reduced, and he became extremely hoarse. The cough and hæmoptoe left him in the course of six weeks, under the use of an oily emulsion, with squills and digitalis; but the hoarseness continues intractable. His 'big manly voice,' for he was a strong man, has sunk into something smaller than 'childish treble.'—If any of the readers of these observations should have found a successful remedy for hoarseness, where the voice is reduced to a whisper after a cough, their imparting it, through the medium of our Journal, may be productive of benefit.—In another case of cough, in which hæmoptoe occurred in considerable quantity, the patient, an elderly thin man, getting rid of twelve ounces of blood at a time, sometimes as often as every six hours for two days together, relief was at first obtained by a draught of Infus. Rosæ, Magnes. Sulphat, and Tinct. Digitalis, every four or six hours, a blister being applied on the chest. But some days after the complaint was seemingly removed, the bleeding returned with such violence, as to threaten dissolution, when the following draught produced a speedy and most salutary effect.

℞. Plumbi Super-acet. gr. iij.

Infus. Digitalis, ℥ j.

Tinct. Opii, gr. v. M. ft. haustus 6ta quaque horâ sumendus.

Nausea and pain in the bowels occurred after the third draught, but the bleeding stopped, and the man is at this moment quite well, though probably doomed to another not very distant attack of his dangerous malady. In youth there is some chance for a man who has escaped from such a complaint, to become strong after it, and the disposition to the disease may be destroyed; but after the middle period of life, there is less hope for any such favourable change of constitution, and it becomes imperious that an individual thus unhappily circumstanced should be most careful in avoiding the exciting causes of the disease. In this affection, as in many others, no certain routine of practice can be laid down; it is a very common practice to take away blood, besides what the patient is pumping up. It will be gratifying to the writer of this article to obtain the opinion of some of his experienced brethren on this subject; also on the effects of emetics in hæmoptoe, as recommended by a German physician, whose name he at present forgets.

In

In the case of a girl aged fourteen, of slender make, and who has not menstruated, affected with *chorea* to a considerable degree, a fair trial has been given to active purgatives for two months, without the slightest alleviation of symptoms; but with manifest reduction of strength and flesh. This plan of treatment was enforced soon after the disease first appeared. For the last month it has been abandoned as hopeless, and steel and zinc have been taken, and the shower and cold bath used, but hitherto without effect.

Amongst the acute diseases which have lately occurred, were cases of cynanche tonsillaris, measles, and small-pox. In two bad cases of measles, one of which died, the mother affirms, that both the children had been affected with the complaint before. One of the cases of small-pox occurred in a boy aged fourteen, whose face exhibited one mass of scab, the disease being far advanced before a physician was called in. He recovered, contrary to expectation. The tide of plebian prejudice still sets strong against vaccination; and its cause is not aided by the ill-timed zeal of those of its advocates who go about, lancet in hand, almost compelling poor ignorant women to have their infants inoculated with cow-pock matter. The great work will proceed; but its progress will not be accelerated by beginning with the uninformed portion of our population.

In the Westminster Hospital, a very large Inguinal Aneurism has been the subject of an operation performed by Mr. White. One ligature alone was used in the tying of the iliac artery. Mr. White, in this operation, avoided the division of the lower edges of the internal, oblique, and transversalis muscles, which mode of operation had, in two cases, left the parts divided so extremely weak, that a considerable hernial tumour was the consequence, which required for its support the constant application of a bandage. The fibres of these muscles were divided down to the peritoneum, at a distance from their lower transverse edges, and Mr. White had no difficulty in securing the artery. This operation, although performed on a very unhealthy patient, has been attended with unusual good results. The thermometer, placed in the ham of the limb the day subsequent to the tying of the vessel, was at 95, which was the temperature of the rest of the body. The man was enabled to walk, without the assistance of crutches, three weeks afterwards; and suffered, in a comparatively small degree, those irksome sensations of numbness, and want of motion in the extremity, which ordinarily have occurred in other cases.

An operation, we believe, new in the annals of surgery, has also been undertaken by Mr. White, on a patient, at Cambridge. The history of this case, we have been told, is, that, from a blow received six years ago on the lower jaw, there had been, during that time, a slow accumulation of diseased structure: the eye was considerably protruded from its orbit, and the health of the patient had suffered very materially. Mr. White, from an examination of the parts, suspected the jaw to be dead from the symphisis to its articulation with the head, and undertook its entire removal. This was accomplished by dividing, with Hey's saws, the bone into three portions, after separating the external parts. Not a vessel was tied during the operation. The bone was entirely dead; and the patient, who, during many years, could barely separate the jaws to admit even a spoon with his food, can now receive and masticate, with the remaining under half, solid animal and other nutriment without the least difficulty. This patient, we have also been informed, had not slept in a horizontal position for the last five years, the accumulation of the secretions from the diseased parts producing, when recumbent, so much irritation in the parts about the throat, as to threaten suffocation. Since the operation, all this distress has ceased, the face has acquired its original size, and the general health of the patient has been established. We trust Mr. White will give us an ample detail of the history of this disease, and its treatment.

